# Supplementary material for: Aging-linked systemic lipid signature is reprogrammed by caloric restriction in rhesus monkeys
Source: Mol Syst Biol. 2025 Dec 10;22(2):281–305. doi: 10.1038/s44320-025-00177-3 (PMC12864875; doi:10.1038/s44320-025-00177-3)
Supplement: Supplementary file 1 — Appendix [file 44320_2025_177_MOESM1_ESM.pdf]

Appendix

Aging-linked systemic lipid signature is reprogrammed by caloric restriction in rhesus monkeys

Salma I. Abou Elhassan[1], Josef P. Clark[2,3], Di Kuang[2], Timothy W. Rhoads[4], Ricki J. Colman[5,6], Joshua J. Coon[1,7,8,9], Rozalyn M. Anderson[2,3], and Katherine A. Overmyer[1,7,9]

[1] Department of Biomolecular Chemistry, University of Wisconsin-Madison, Madison, WI 53706, USA
[2] Department of Medicine, University of Wisconsin-Madison, Madison, WI 53705, USA
[3] Geriatric Research, Education, and Clinical Center, William S. Middleton Memorial Veterans Hospital, Madison, WI, 53705 USA
[4] Department of Nutritional Sciences, University of Wisconsin-Madison, Madison, WI 53706, USA
[5] Wisconsin National Primate Research Center, University of Wisconsin-Madison, WI 53715, USA
[6] Department of Cell and Regenerative Biology, University of Wisconsin-Madison, Madison, WI 53705, USA
[7] Morgridge Institute for Research, Madison, 53715, WI, USA
[8] Department of Chemistry, University of Wisconsin-Madison, Madison, WI 53706, USA
[9] National Center for Quantitative Biology of Complex Systems, Madison, WI 53706, USA

*Correspondence: **Katherine A. Overmyer** (kovermyer@wisc.edu), **Rozalyn M. Anderson** (rozalyn.anderson@wisc.edu)

Table of Contents

Tables……………………………………………………………………………………………3

Appendix Table S1. Capillary LC gradient ……………………………………………………3

Appendix Table S2. Concentrations of C^{13} N^{15} amino acid and lipid standard mixtures……………4

Appendix Table S3. Lipid Classes and corresponding abbreviations used in this study………………5

Figures……………………………………………………………………………………………6

Appendix Figure S1. Concentrations of C^{13} N^{15} amino acid and lipid standard mixtures…………………6

Appendix Figure S2. Evaluation of the combined metabolomics and lipidomics method on human NIST 1950 plasma and monkey serum samples ……………………………………………7

Appendix Figure S3. Longitudinal lipidomic and metabolomic differences between caloric-restricted (CR) and control-fed (C) monkeys………………………………………………………8

Appendix Figure S4. Longitudinal lipidomic and metabolomic differences between male (M) and female (F) monkeys……………………………………………………………………9

Appendix Figure S5. Age and age-associated differences of lipid abundance in rhesus monkeys…..10

Appendix Table S1. Capillary LC gradient. The table summarizes the solvent gradient and flow rate profile for the liquid chromatography (LC) method. Mobile phase B consisted of isopropanol:acetonitrile (9:1, v/v) with 10 mM ammonium formate and 0.2% formic acid, and mobile phase A consisted of 0.2% formic acid in water.

**Time (min)**	**Flow Rate (μL/min)**	**B (%)**
0	80	0
1	80	0
7	80	100
11	80	100
11.25	100	100
12	100	0
13.9	100	0
14	80	0

Appendix Table S2. Concentrations of SPLASH and Cell Free C^{13} N^{15} Amino Acid internal standard mixtures. The table lists the concentrations (in μM) of individual components included in the internal standard mixtures used for evaluation of the method's limit of detection and included in the analysis of the monkey serum samples for quality assessment.

Internal Standard	Concentration (mM)
Alanine	100
Arginine	10
Aspartamine	20
Aspartic acid	60
Cholesteryl ester (CE) 18:1(d7)	0.24993
Cystine	20
Diglycerides (DG) 15:0/18:1(d7)	0.014966
Glutamic acid	40
Glutamine	20
Histidine	5
Isoleucine	30
Leucine	45
Lysophosphatidylcholine (LPC) 18:1(d7)	0.045014
Lysophosphatidylethanolamine (LPE) 18:1(d7)	0.010069
Lysine	15
Methionine	10
Phosphatidic acid (PA) 15:0/18:1(d7)	0.010001
Phosphatidylcholine (PC) 15:0/18:1(d7)	0.199971
Phosphatidylethanolamine (PE) 15:0/18:1(d7)	0.007454
Phosphatidylglycerol (PG) 15:0/18:1(d7)	0.034947
Phenylalaine	16
Phosphatidylinositol (PI) 15:0/18:1(d7)	0.010034
Proline	20
Phosphatidylserine (PS) 15:0/18:1(d7)	0.005019
Serine	35
Sphingomyelin (SM) 18:1/18:1(d9)	0.040102
Triglyceride (TG) 15:0/15:0/18:1(d7)	0.064995
Threonine	35
Tryptophan	20
Tyrosine	10
Valine	40

Appendix Table S3. Lipid classes and corresponding abbreviations used in this study. The table lists all lipid classes identified in this study along with their corresponding abbreviations as used in figures and tables throughout the manuscript.

**Lipid Class**	**Abbreviation**
Acylcarnitines	AC
Alkanyl-Diacylglycerol	Alkanyl-DG
Alkanyl-Triacylglycerol	Alkanyl-TG
Alkenyl-Diacylglycerol	Alkenyl-DG
Alkenyl-Triacylglycerol	Alkenyl-TG
Cholesteryl ester	CE
CeramideNS	Cer[NS]
Diacylglycerol; Diglyceride	DG
Dimethyl Phosphatidylethanolamine	PE-NMe2
Hexosyl CeramideNS	HexCer[NS]
Lysophosphatidylcholine	LysoPC
Lysophosphatidylethanolamine	LysoPE
Lysophosphatidylglycerol	LysoPG
Lysophosphatidylinositol	LysoPI
Lysosphingomyelin	LysoSM
Phosphatidylcholine	PC
Phosphatidic acid	PA
Phosphatidylglycerol	PG
Phosphatidylethanolamine	PE
Phosphatidylinositol	PI
Phosphatidylserine	PS
Plasmanyl Phosphatidycholine	Plasmanyl-PC/ O-PC
Plasmanyl Phosphatidylethanolamine	Plasmanyl-PE/ O-PE
Plasmenyl Phosphatidycholine	Plasmenyl-PC/ P-PC
Plasmenyl Phosphatidylethanolamine	Plasmenyl-PE/ P-PE
Sphingomyelin	SM
Sphingosine-1-Phosphate	S1P
Triacylglycerol; Triglyceride	TG

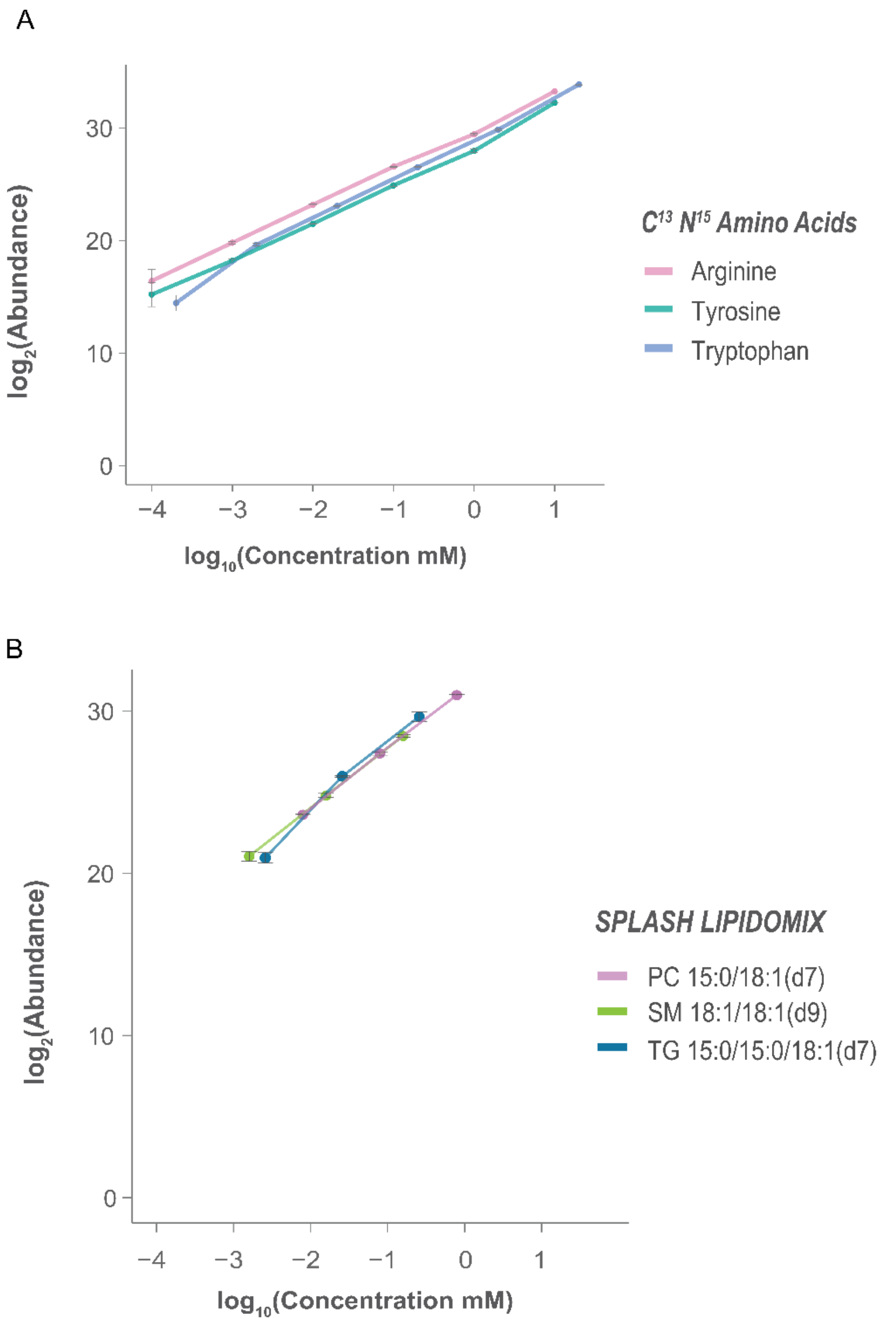

Appendix Figure S1. Loading amounts of spiked in isotopically labeled internal standards, amino acids (A), and Avanti SPLASH LIPIDOMIX mixture (B), into NIST1950 extracts. Dots represent the mean of log2 abundance of each loading amount (*n*=3), error bars show standard deviation.

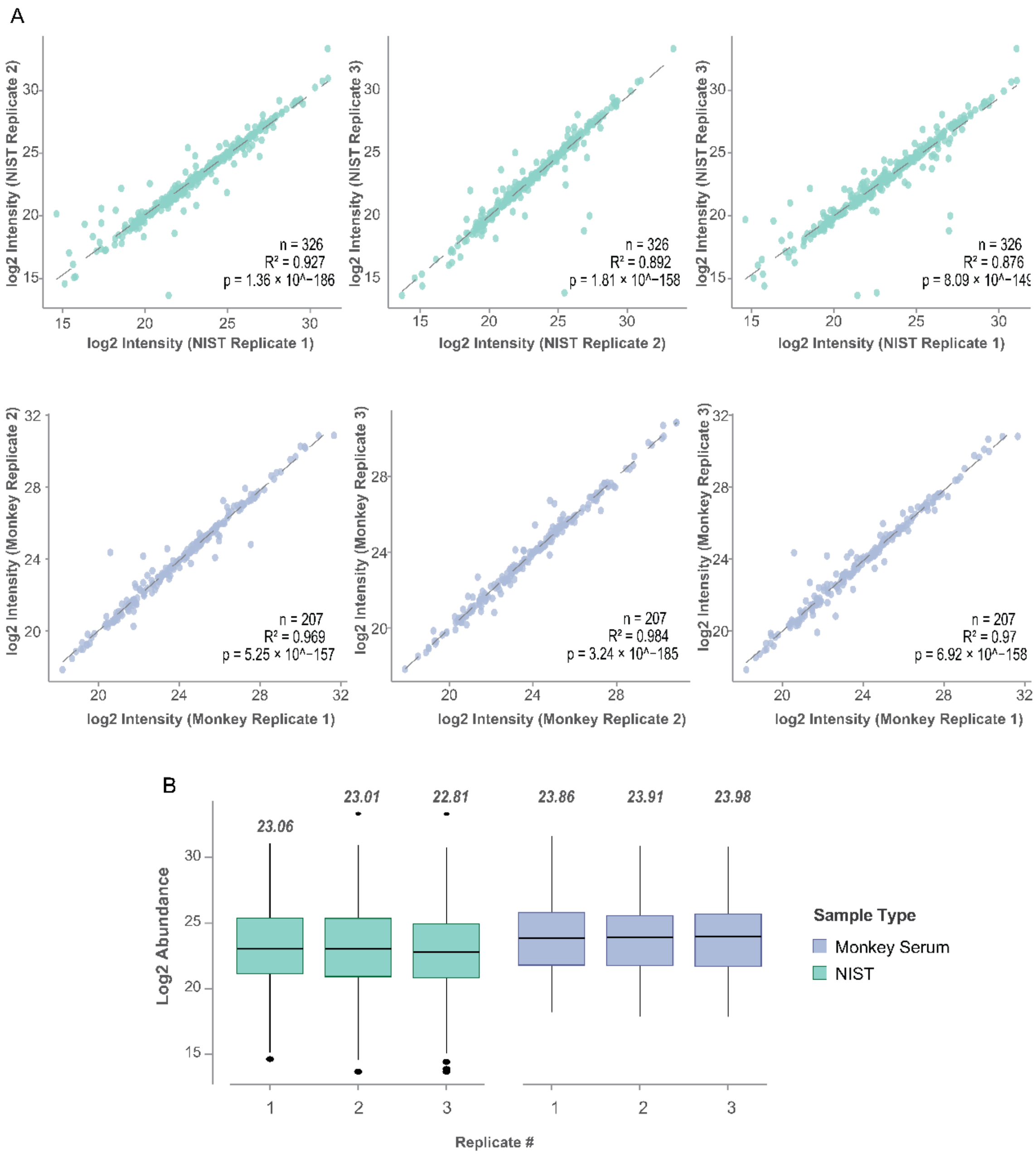

Appendix Figure S2. Evaluation of the combined metabolomics and lipidomics method on human NIST1950 plasma and monkey serum samples. (A) Scatter plot highlights the reproducibility of three technical replicates from NIST1950 (in green) and pooled monkey serum samples (in purple); each dot represents the abundance of biomolecule features. The reproducibility was assessed by computing Pearson's correlations between log-transformed intensity values of technical replicates (log_2-transformed). The coefficient of determination (R^2) was obtained by squaring the Pearson correlation coefficient, and statistical significance was evaluated using a two-sided Pearson correlation test. The number of biomolecules (n), R^2, and p-value (p) are reported for each plot. **(B)** Boxplots show log2 abundance of features across replicates of monkey serum (purple) and Human NIST1950 plasma (green). For each boxplot, the middle horizontal line is the median, box margins are first and third quartiles, with vertical lines extending ± 1.5-times the interquartile range, and each dot represents the sample's abundance.

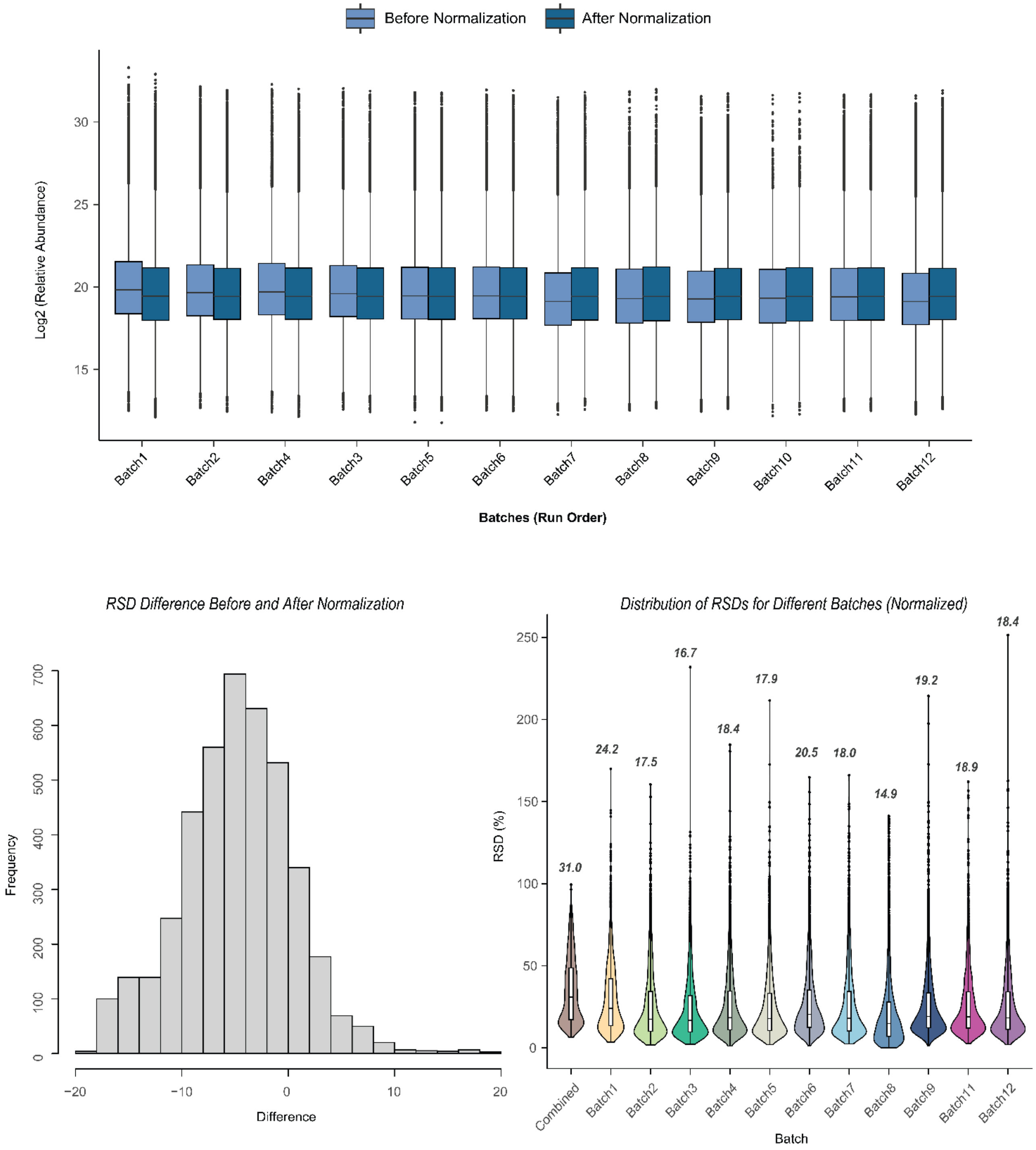

Appendix Figure S3. Evaluation of normalization across batches of monkey plasma samples. (**A**) Boxplots show the distribution of log_2-transformed relative abundances before and after normalization across 12 analytical batches. **(B)** Histogram shows feature-wise relative standard deviation (RSD) differences before *vs.* after normalization. Most features shift leftward, indicating reduced variance following normalization. **(C)** Violin plot shows the distribution of RSD% values in the quality control monkey serum samples that were run alongside study samples, for each batch and across all batches combined, after normalization.

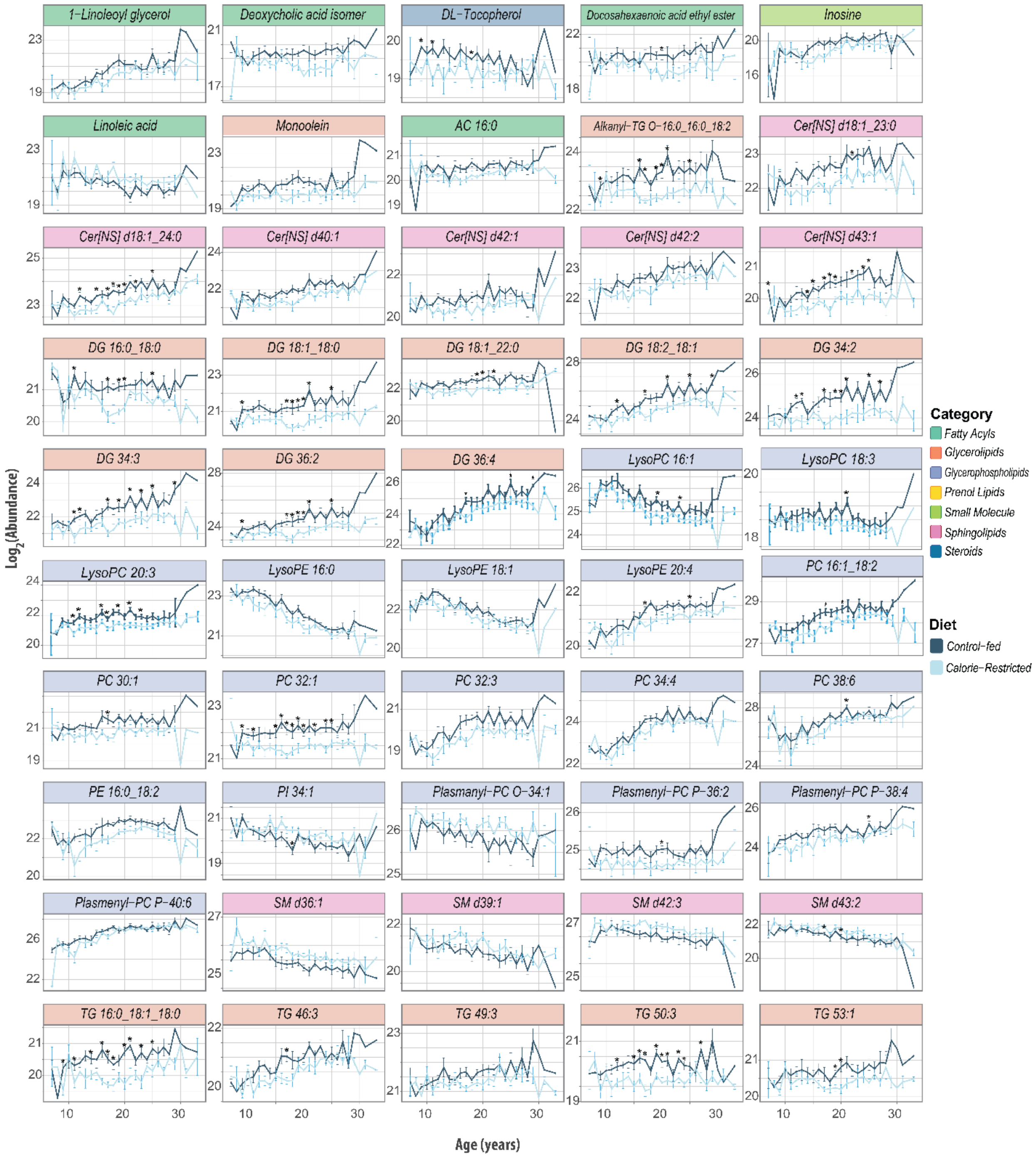

Appendix Figure S4. Longitudinal lipidomic and metabolomic differences between caloric-restricted (CR) and control-fed (C) monkeys. Biomolecule abundances significantly associated with diet are plotted over the lifespan of C and CR monkeys; dots show the mean abundance, error bars show the standard error of the mean, and stars indicate adjusted *p*-value < 0.05 using Welch's unpaired *t*-test.

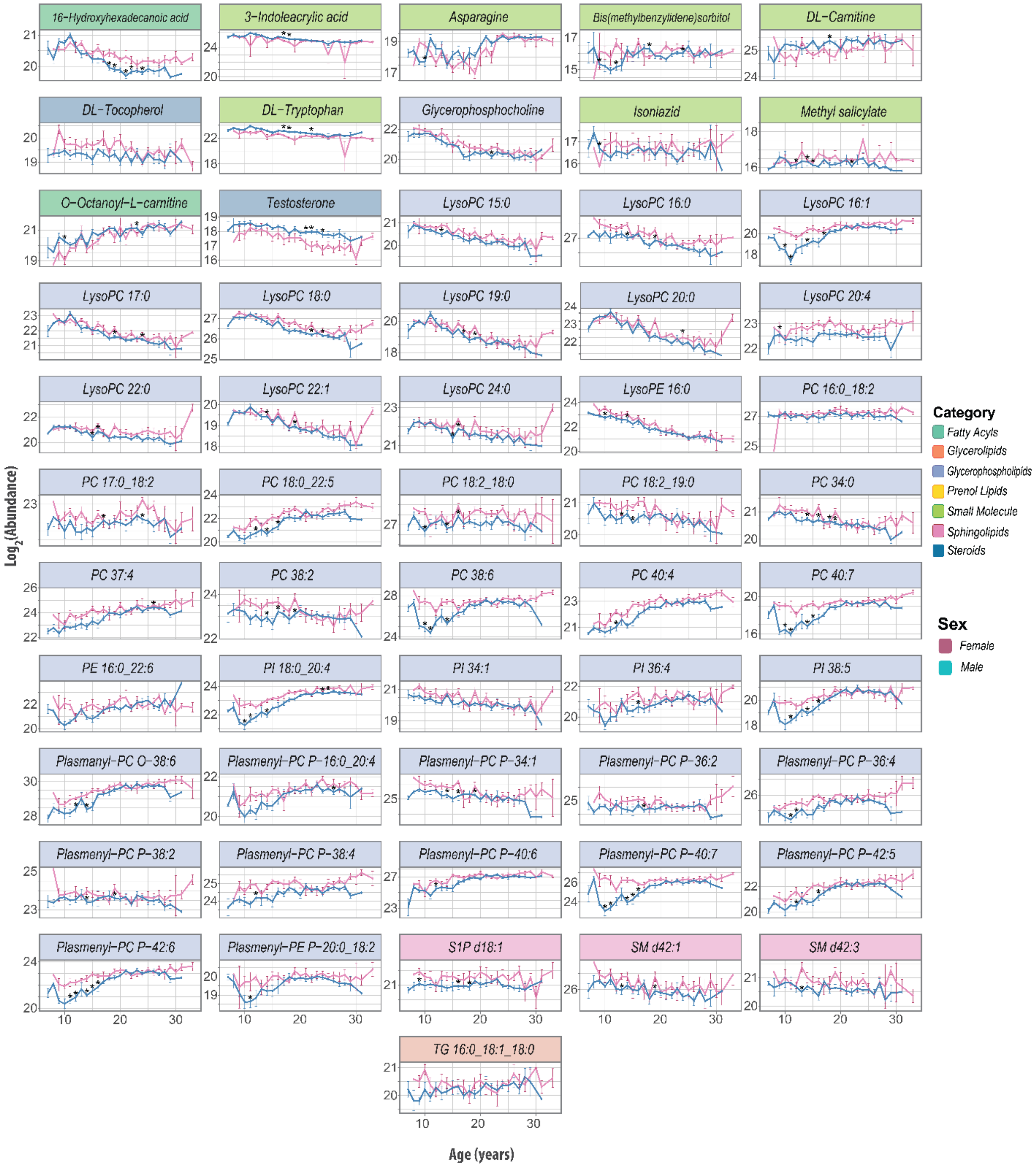

Appendix Figure S5. Longitudinal lipidomic and metabolomic differences between male (M) and female (F) monkeys. Biomolecule abundances significantly associated with sex are plotted over the lifespan of M and F monkeys; dots show the mean abundance, error bars show the standard error of the mean, and stars indicate adjusted *p*-value < 0.05 using Welch's unpaired *t*-test.

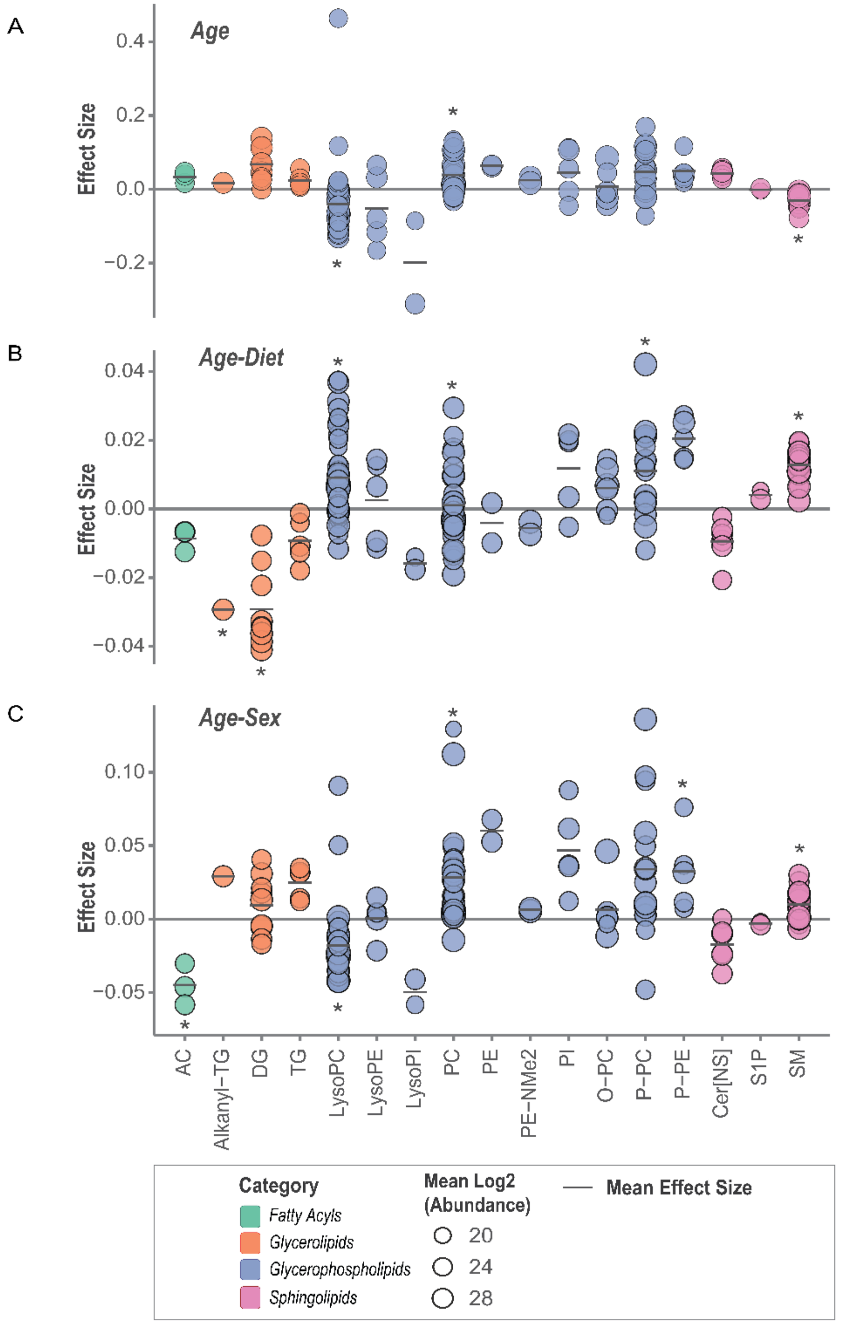

Appendix Figure S6. Age and age-associated differences of lipid abundance in rhesus monkeys. (A) Effect sizes based on the linear regression results for (Younger *vs.* Older monkeys) across lipid classes. **(B)** Effect sizes based on the linear regression results for age-diet interactions, and **(C)** Effect sizes based on the linear regression results for age-sex interactions. Effect sizes are plotted for each lipid class and colored by lipid category. Each point represents a lipid species that is colored and grouped by lipid category. Point size is proportional to the mean log2 abundance of the related feature/lipid species across all samples. Horizontal bars indicate the mean of effect sizes within each class. Negative values denote higher abundance in Females, while positive values denote higher abundance in Males. Lipid classes identified as significantly enriched or depleted by enrichment analysis are denoted with stars.
