# Supplementary material for: Aging-linked systemic lipid signature is reprogrammed by caloric restriction in rhesus monkeys
Source: Mol Syst Biol. 2025 Dec 10;22(2):281–305. doi: 10.1038/s44320-025-00177-3 (PMC12864875; doi:10.1038/s44320-025-00177-3)
Supplement: Supplementary file 9 — Expanded View Figures [file 44320_2025_177_MOESM9_ESM.pdf]

Expanded View Figures

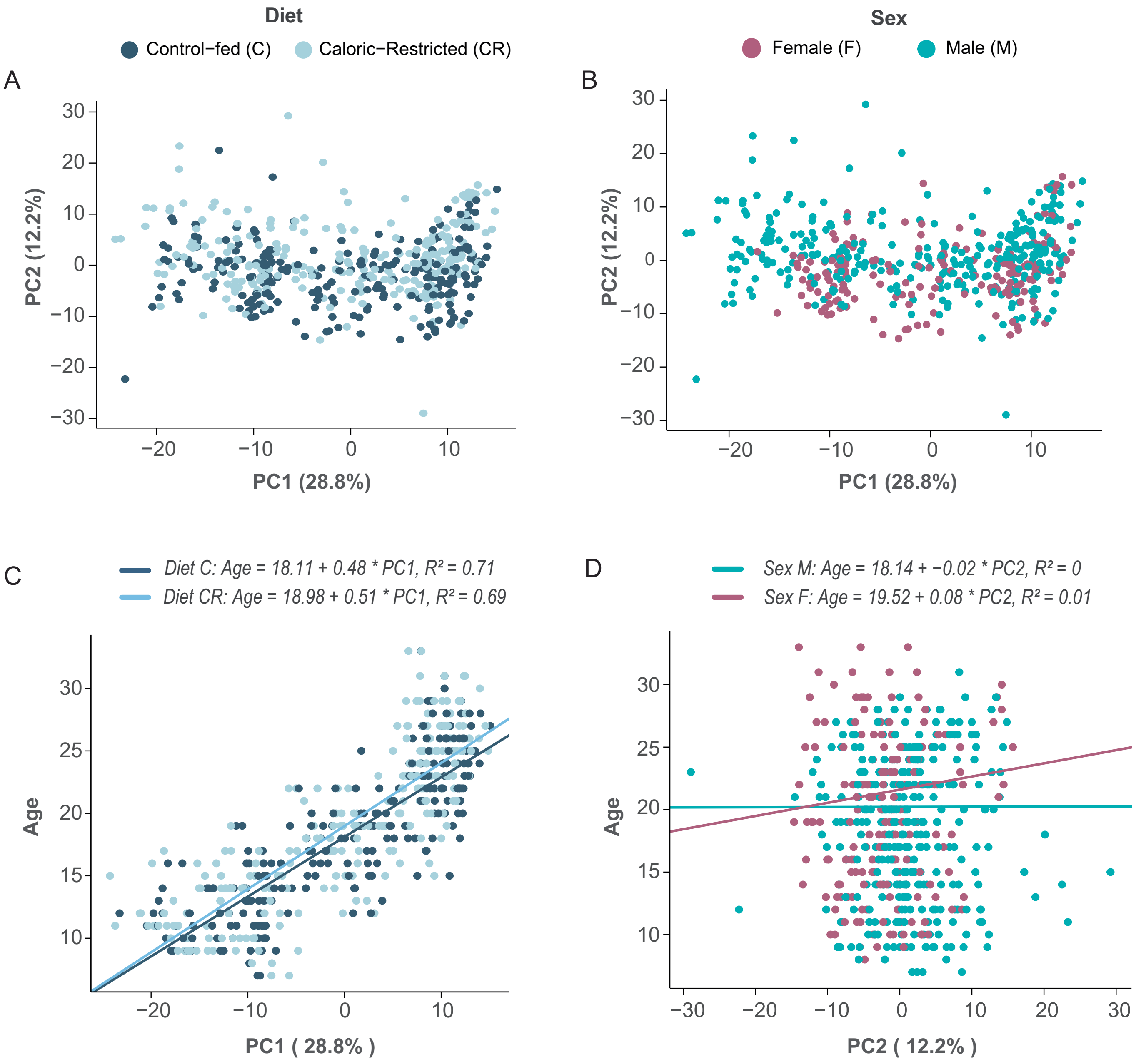

Figure EV1. Evaluating the impact of diet and sex in the principal component analysis (PCA) of the metabolite data.

(**A**, **B**) PCA of study samples using quantitative values from omics data (lipids and small molecules, $\log_2$ transformed and centered around 0 for $n = 494$ monkey plasma samples), shows that principal components 1 and 2 capture 28.8 and 12.2%, respectively, of the variance between monkey plasma samples; dots colored based on the type of diet applied to the monkeys (**A**) or sex of the studied monkeys (**B**). (**C**) The scatter plot shows principal component (PC) 1 relative to the age of monkeys to evaluate the effect of caloric restriction (CR) on PC1. (**D**) The scatter plot shows PC2 values relative to the age of monkeys to evaluate the effect of sex on PC2. Data information: Linear regression lines were fitted separately for each group in (**C**, **D**), with the coefficient of determination (R^2) calculated for each regression model. The regression equations, including slope and intercept, are displayed on each plot.

 © The Author(s)

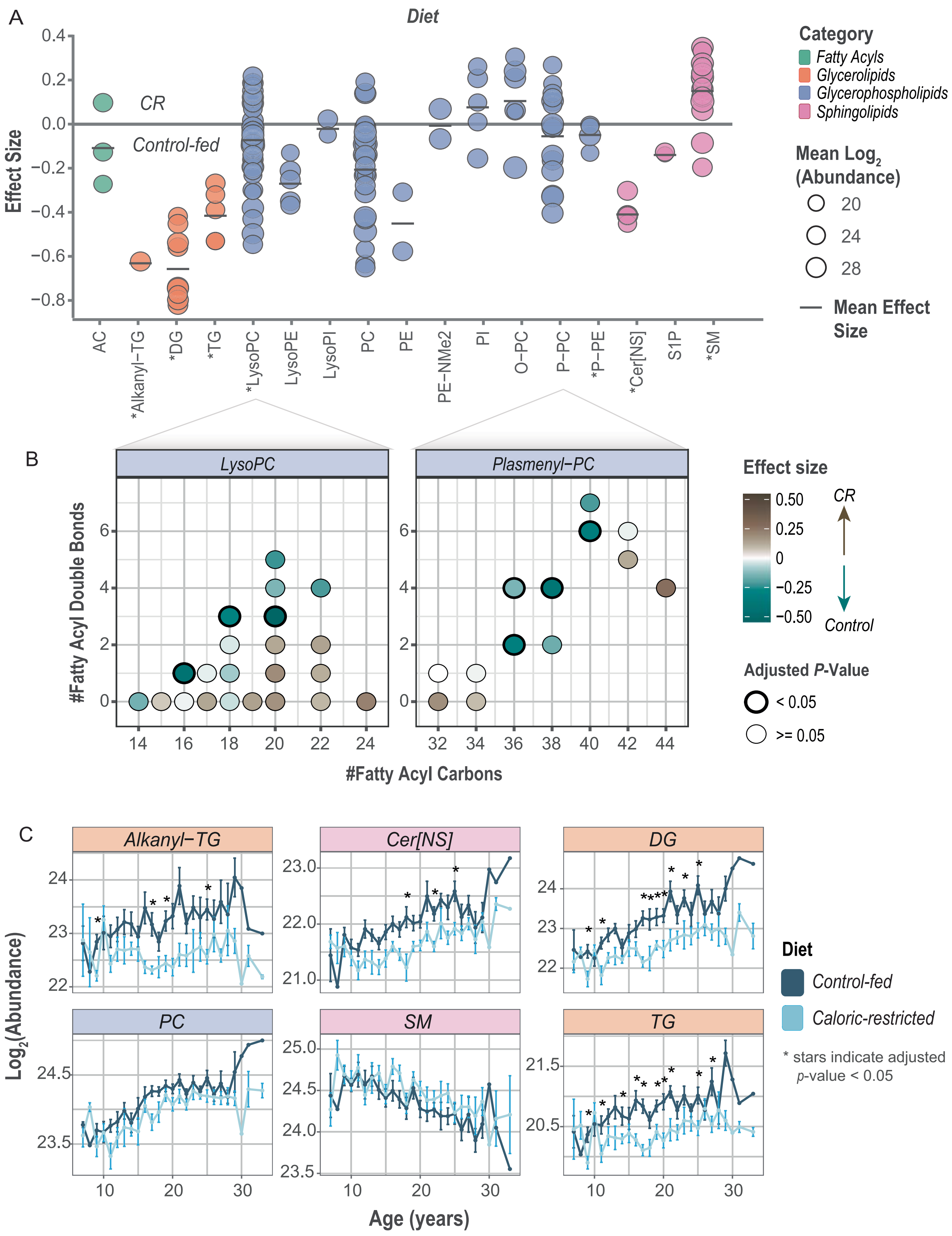
A
Diet
Effect Size
CR
Control-fed
AC
*Alkanyl-TG
*DG
*TG
*LysoPC
LysoPE
LysoPI
PC
PE
PE-NMe2
PI
O-PC
P-PC
*P-PE
*Cer[NS]
S1P
*SM
Category
Fatty Acyls
Glycerolipids
Glycerophospholipids
Sphingolipids
Mean Log2 (Abundance)
20
24
28
Mean Effect Size
B
LysoPC
Plasmenyl-PC
#Fatty Acyl Double Bonds
#Fatty Acyl Carbons
Effect size
CR
Control
Adjusted P-Value
< 0.05
>= 0.05
C
Alkanyl-TG
Cer[NS]
DG
PC
SM
TG
Log2(Abundance)
Age (years)
Diet
Control-fed
Caloric-restricted
* stars indicate adjusted p-value < 0.05

◀ **Figure EV2. Dietary effects on lipid abundance in rhesus monkeys.**

(**A**) Effect sizes (CR vs. control-fed) are plotted for each lipid class and colored by lipid category. Each point represents a lipid species, with point size proportional to the mean $\log_2$ abundance of the related feature/lipid species across all samples. Horizontal bars indicate the mean effect size within each class. Negative values denote lower abundance under CR, while positive values denote higher abundance under CR. Lipid classes identified as significantly enriched or depleted by enrichment analysis are denoted with stars by name. (**B**) Detailed analysis of lysoPC and plasmenyl-PC are plotted by the fatty acyl chain length (number of fatty acyl carbons, x-axis) and the degree of unsaturation on fatty acyl chain (number of fatty acyl double bonds, y-axis). Circle stroke indicates the significance of lipid species based on their adjusted *p* values from the regression models. Circle color represents effect size (CR vs. Control-fed). (**C**) Averaged abundances of lipid classes significantly associated with diet are plotted as a function of age; title colors are based on the lipid category, and dots show the mean abundance. Data information: For (**C**), error bars show the standard error of the mean, and stars indicate adjusted *p* value <0.05 using Welch's unpaired *t*-test.

 © The Author(s)

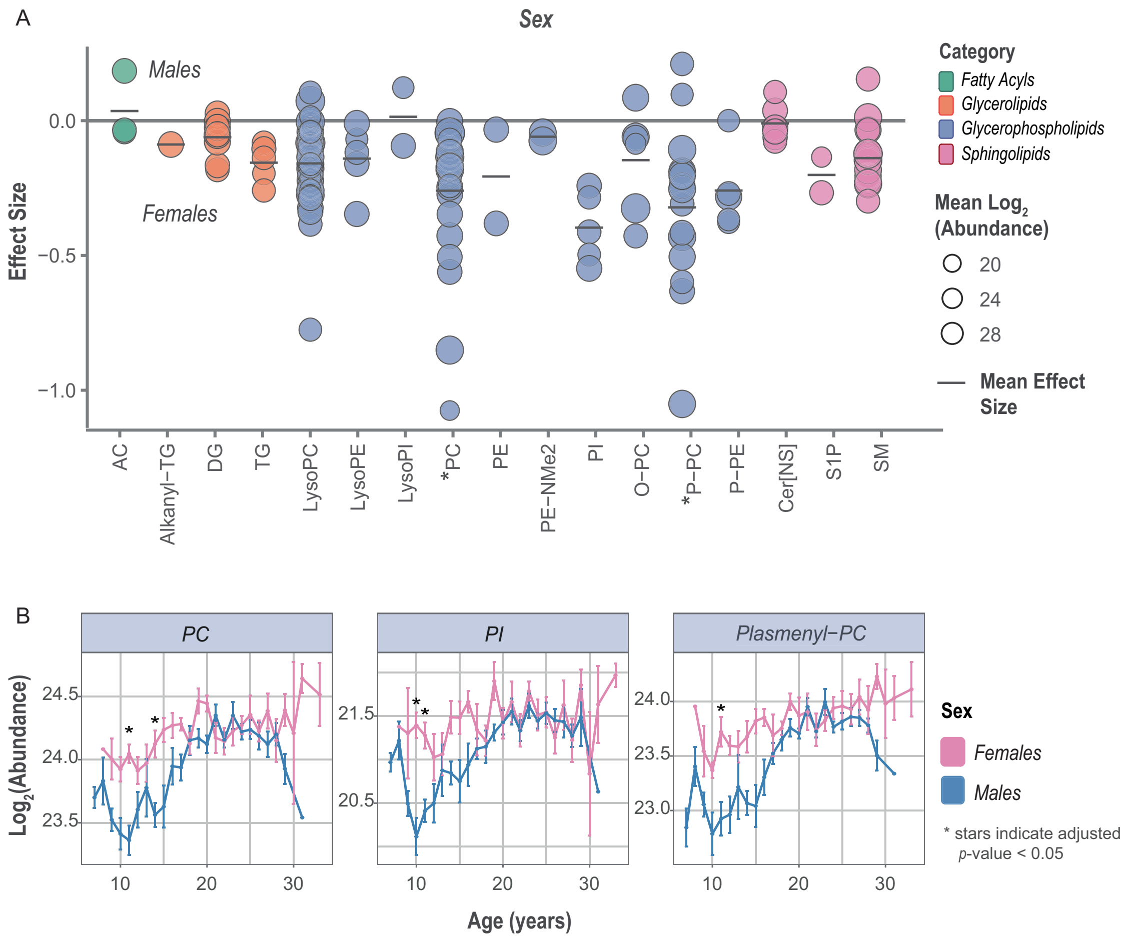

Figure EV3. Sex-specific differences of lipid abundance in rhesus monkeys.

(**A**) Effect sizes (males vs. females) across lipid classes are plotted and colored by lipid category. Each point represents a lipid species, with point size proportional to the mean log2 abundance of the related feature/lipid species across all samples. Horizontal bars indicate the mean effect size within each class. Negative values denote higher abundance in Females, while positive values denote higher abundance in Males. Lipid classes identified as significantly enriched or depleted by enrichment analysis are denoted with stars by name. (**B**) Averaged abundances of lipid classes significantly associated with sex are plotted as a function of age; title colors are based on the lipid category, dots show the mean abundance, and stars indicate adjusted *p* value <0.05. Data information: For (**B**), error bars show the standard error of the mean, and stars indicate adjusted *p* value <0.05 using Welch's unpaired *t*-test.

© The Author(s)
